# Supplementary figures and images for: Genetic ablation of Cullin-RING E3 ubiquitin ligase 7 restrains pressure overload-induced myocardial fibrosis
Source: PLoS One. 2020 Dec 22;15(12):e0244096. doi: 10.1371/journal.pone.0244096 (PMC7755222; doi:10.1371/journal.pone.0244096)

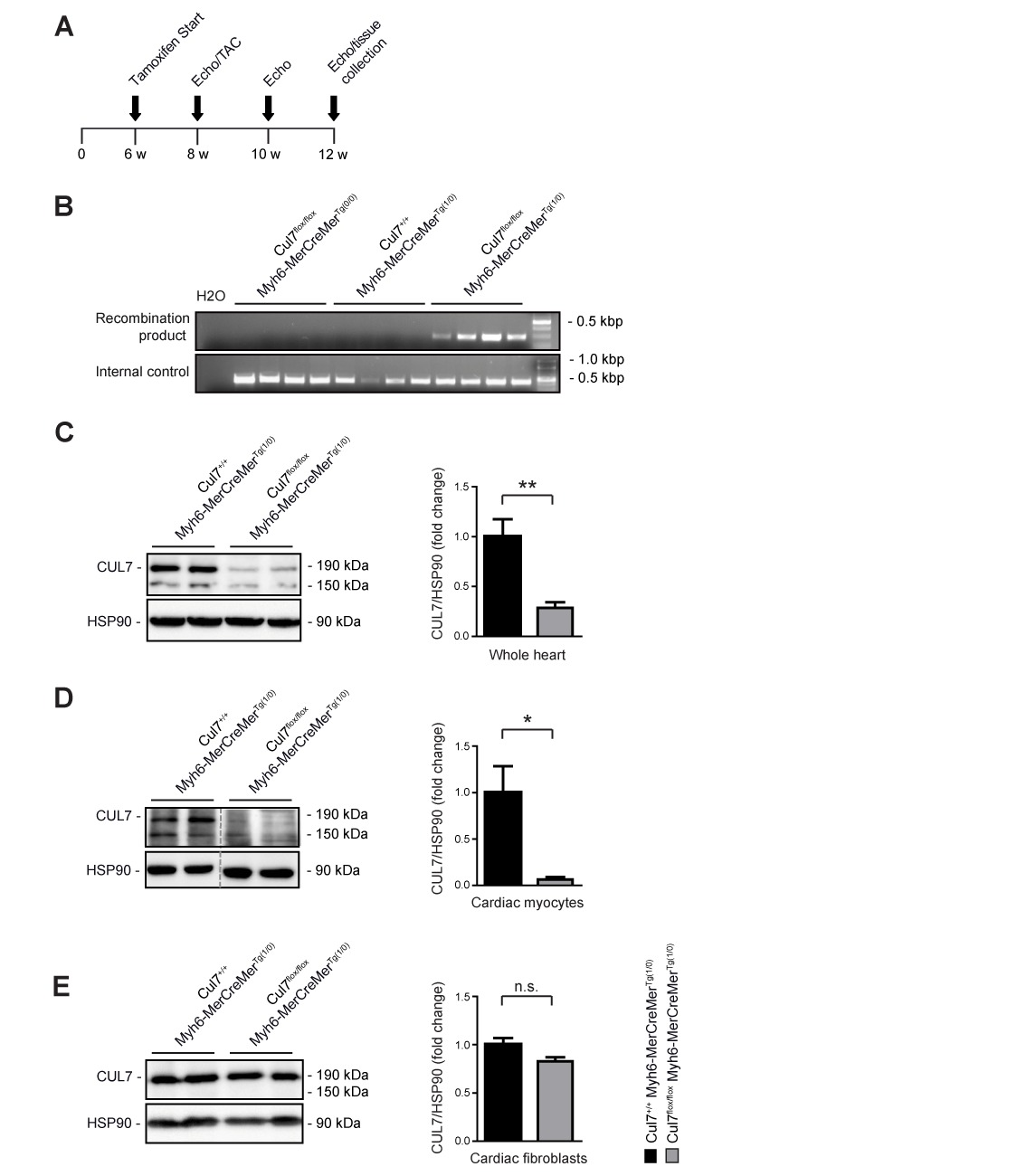

Supplement: S1 Fig — (A) Experimental strategy and timeline. (B) For checking recombination efficacy, a recombination PCR was performed followed by gel electrophoresis. Events of Cul7 allele recombination were only detectable in Cul7flox/flox Myh6-MerCreMerTg(1/0) mice (lanes 10–13), when compared to Cul7+/+ Myh6-MerCreMerTg(1/0) (lanes 6–9) and Cul7flox/flox Myh6-MerCreMerTg(0/0) (lanes 2–5). (C) Significant reduction of the Cullin7 protein abundance in whole hearts samples under basal conditions. n = 4 mice/group. (D) Lysates of isolated cardiac myocytes (CM) were subjected to immunoblot analyses for CUL7 protein. HSP90 served as internal control. Representative blot depicting CM-specific CUL7 knockdown and quantification. n = 4 mice/group. (E) Representative immunoblot of non-CM for CUL7 abundance (left) and quantification thereof (right). n = 4 mice/group. Data are shown as mean ± SEM. Unpaired t-test. *p<0.05, **p<0.01. (TIF) [file pone.0244096.s001.tif]

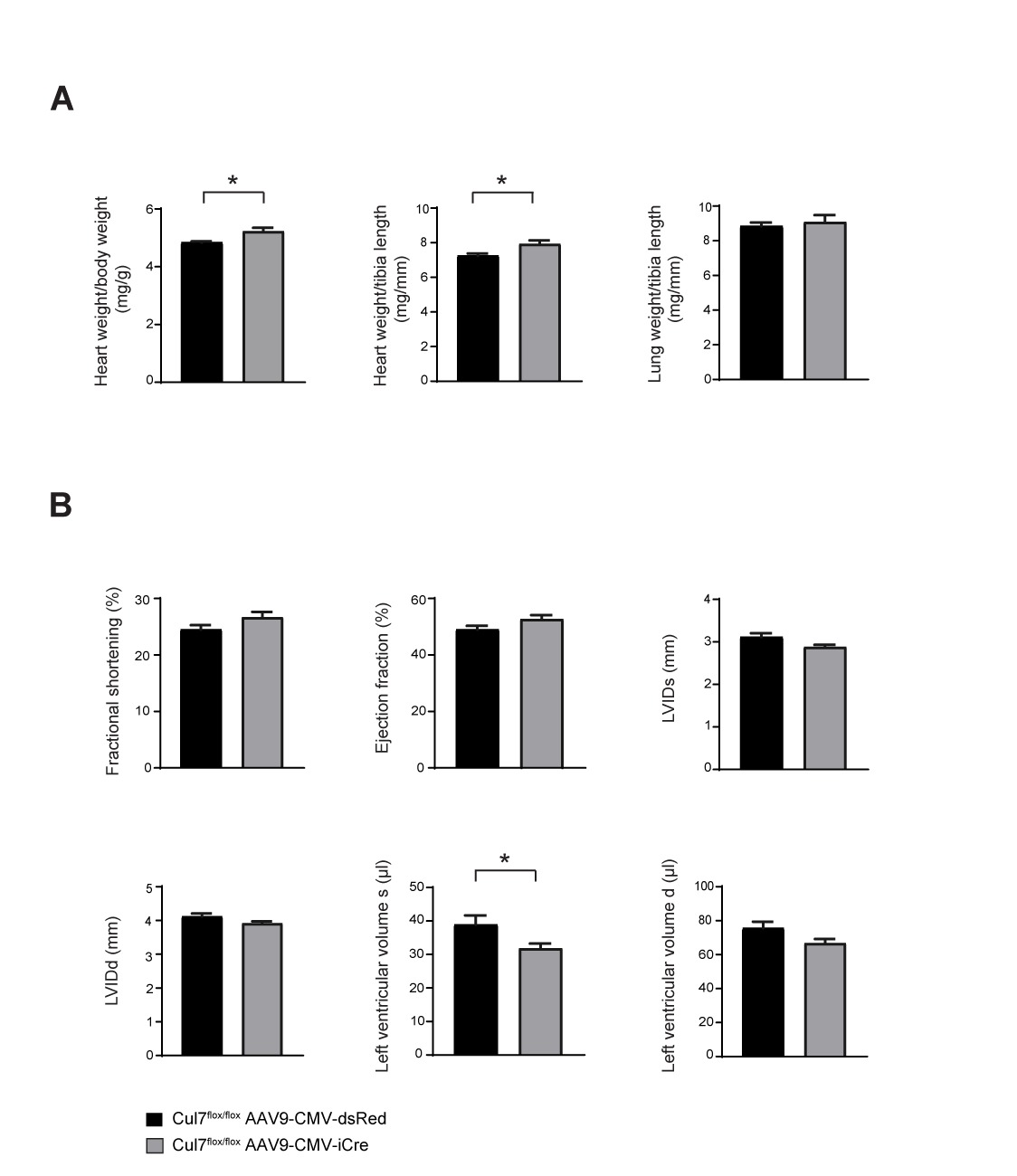

Supplement: S2 Fig — Examination of 8-week-old mice compared to respective control animals injected with AAV9-CMV-dsRed under basal conditions. (A) Screening for cardiac hypertrophy by assessment of heart weight/ body weight-, heart weight/ tibia length-, and lung weight/ tibia length-ratio. n = 5–8 mice/group; *p<0.05, **p<0.01 (unpaired t-test). (B) Echocardiographic assessment of 8-week-old mice under basal conditions concerning measurement of fractional shortening, ejection fraction, left ventricular systolic and diastolic volume as well as left ventricular inner diameter in diastole and systole (LVID d/s). n = 8–11 mice/group; *p<0.05 (unpaired t-test). (TIF) [file pone.0244096.s002.tif]

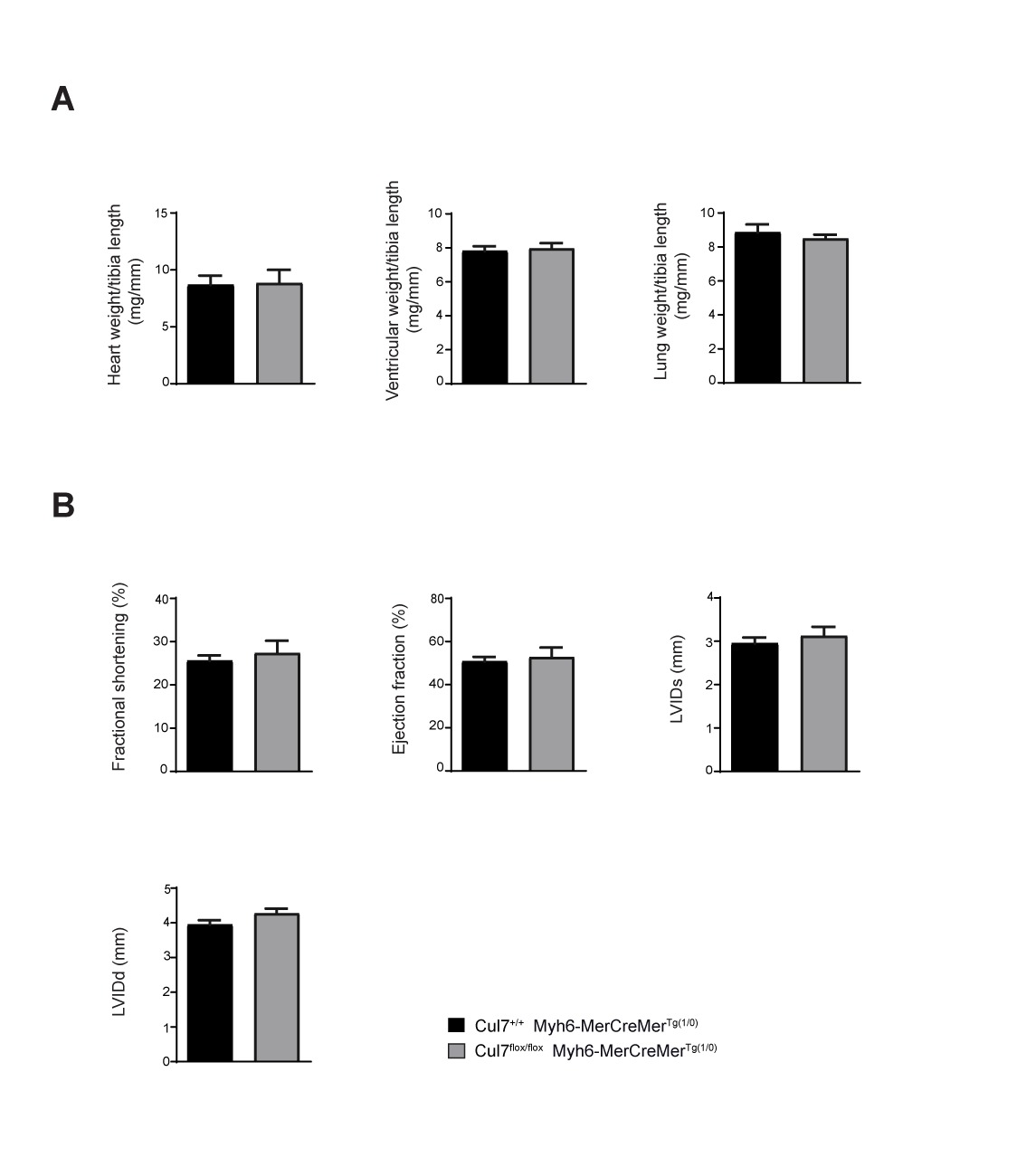

Supplement: S3 Fig — (A) Morphometric assessment of heart weight/tibia length (HW/TL), ventricular weight/tibia length (VW/TL) and lung weight/tibia length (LW/TL) under basal conditions. (B) Functional assessment of fractional shortening, ejection fraction, left ventricular inner diameter in systole and diastole (LVIDs resp. LVIDd) under basal conditions. n = 7–9 mice/group. Data are shown as mean ± SEM. *p<0.05, **p<0.01 (unpaired t-test). (TIF) [file pone.0244096.s003.tif]

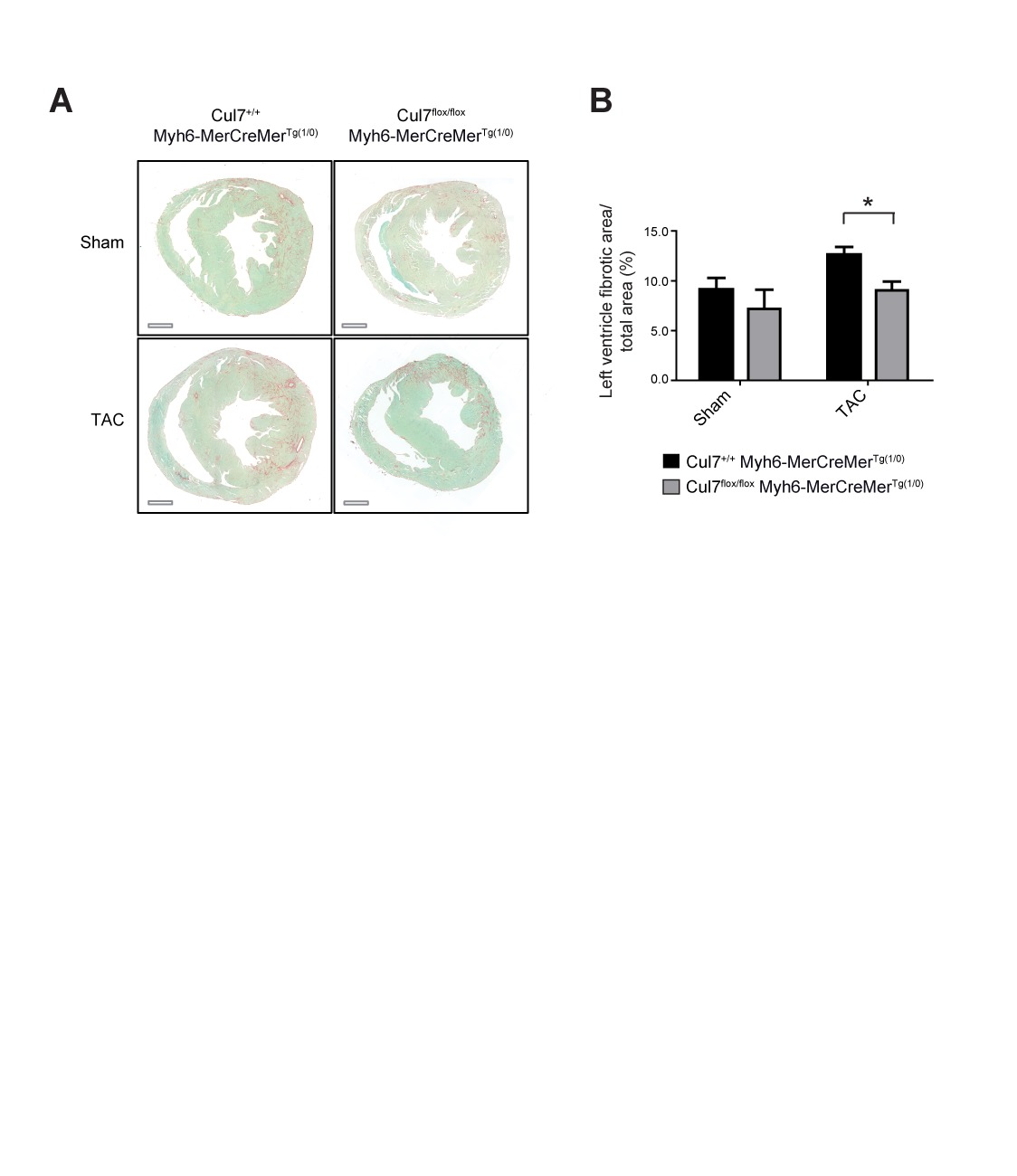

Supplement: S4 Fig — (A) Representative myocardial tissue sections after staining with Sirius Red (for collagen) and Fast Green counterstaining after transverse aortic constriction (TAC; lower panel). Mice were sacrificed after 4 weeks of increased afterload at an age of 12 weeks. Scale bar: 1 mm. (B) Quantification of myocardial fibrosis of sham vs. TAC mice. n = 7–9 mice/group. Data are shown as mean ± SEM. Two-way ANOVA. *p<0.05. (TIF) [file pone.0244096.s004.tif]

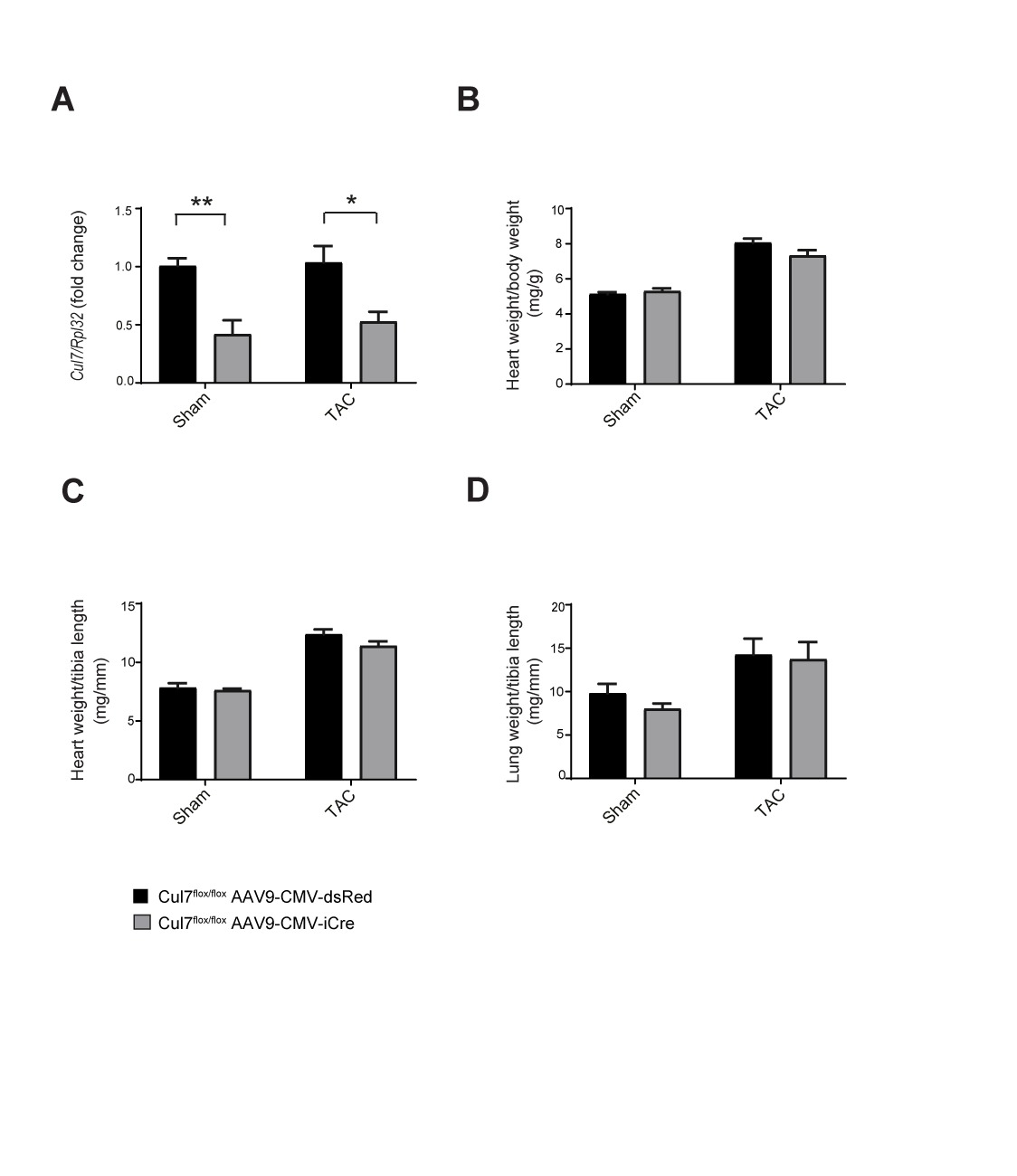

Supplement: S5 Fig — (A) Quantitative qPCR analysis for Cul7 gene expression in whole heart samples 3 weeks after TAC or sham surgery. n = 4–5 mice/group; *p<0.05, **p<0.01 (student’s t-test). Heart weight/ body weight- (B), heart weight/ tibia length- (C) and lung weight/ tibia length-ratios (D). n = 5–6 mice/group; Sham vs. TAC (B-C): highly statistically significant (two-way ANOVA). (TIF) [file pone.0244096.s005.tif]

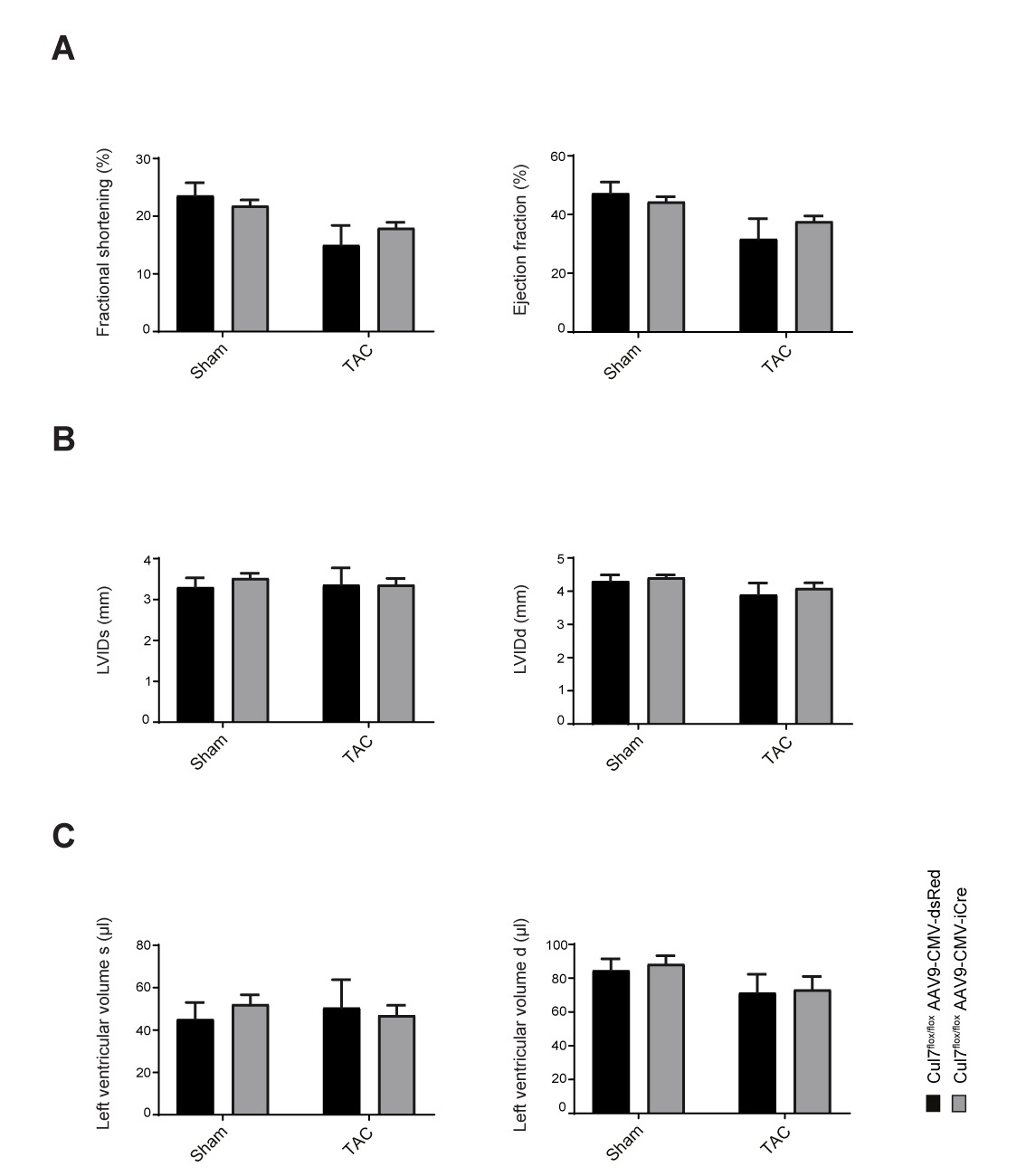

Supplement: S6 Fig — Heart function of mice injected with AVV9 was analysed by pulse-wave Doppler echocardiography upon sham as well as TAC surgery after 3 weeks of increment of afterload. Lane 1–2 sham cohort, lane 3–4 TAC cohort. (A) Functional parameters FS and EF after operation. Sham vs. TAC: highly statistically significant (two-way ANOVA). (B) Left ventricular inner diameter in systole as well as diastole after operation of the sham and the respective TAC cohort. (C) Systolic and diastolic left ventricular volume. n = 4–6 mice/group; Sham vs. TAC: not statistically significant (two-way ANOVA). (TIF) [file pone.0244096.s006.tif]

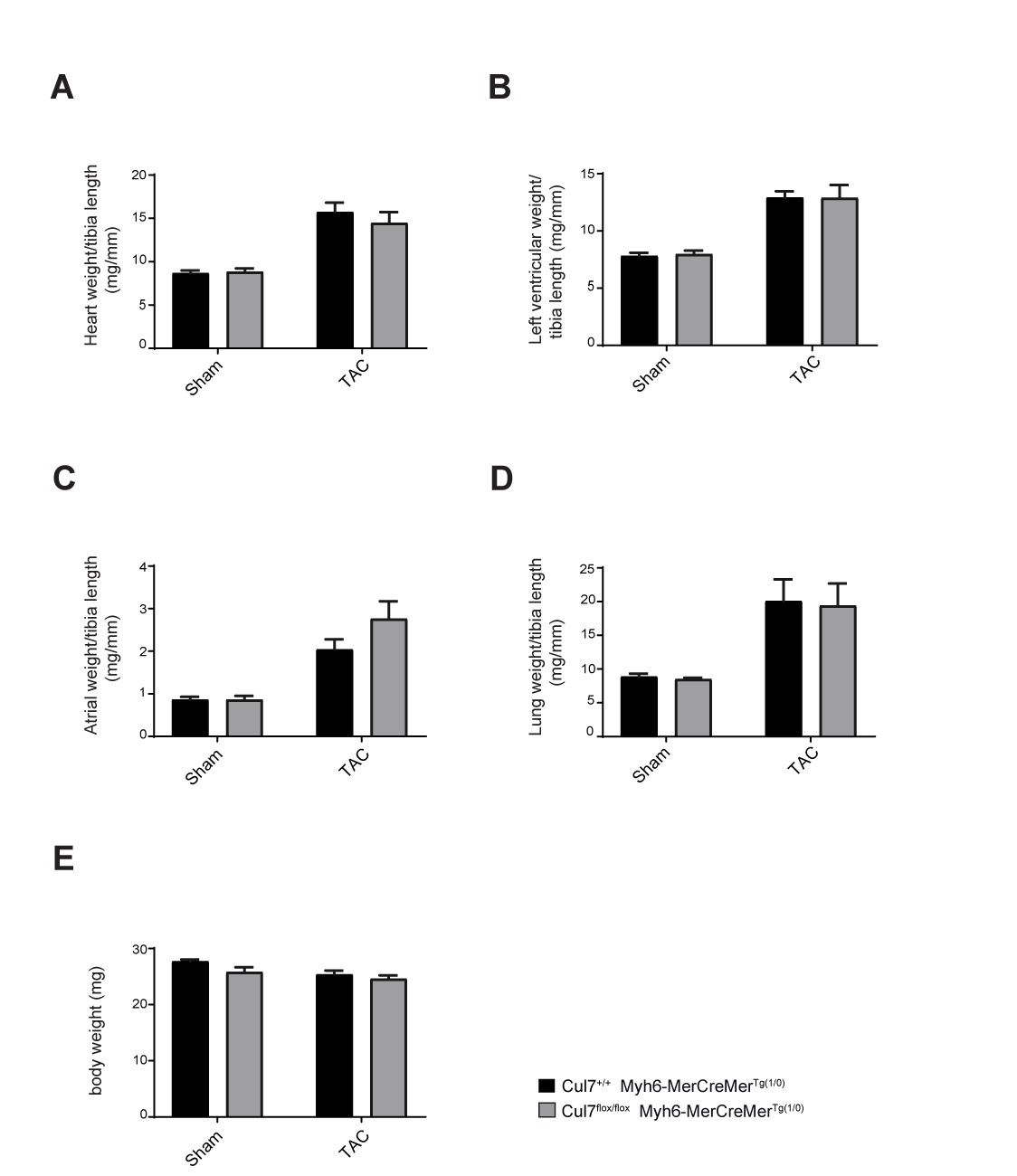

Supplement: S7 Fig — Morphometric assessment of (A) heart weight/tibia length (HW/TL), (B) ventricular weight/tibia length (VW/TL), (C) atrial weight/tibia length (AW/TL), (D) lung weight/tibia length (LW/TL) and (E) body weight under basal conditions (sham) and conditions of increased afterload (TAC). n = 7–9 mice/group. Data are shown as mean ± SEM. *p<0.05, **p<0.01 (two-way ANOVA). (TIF) [file pone.0244096.s007.tif]

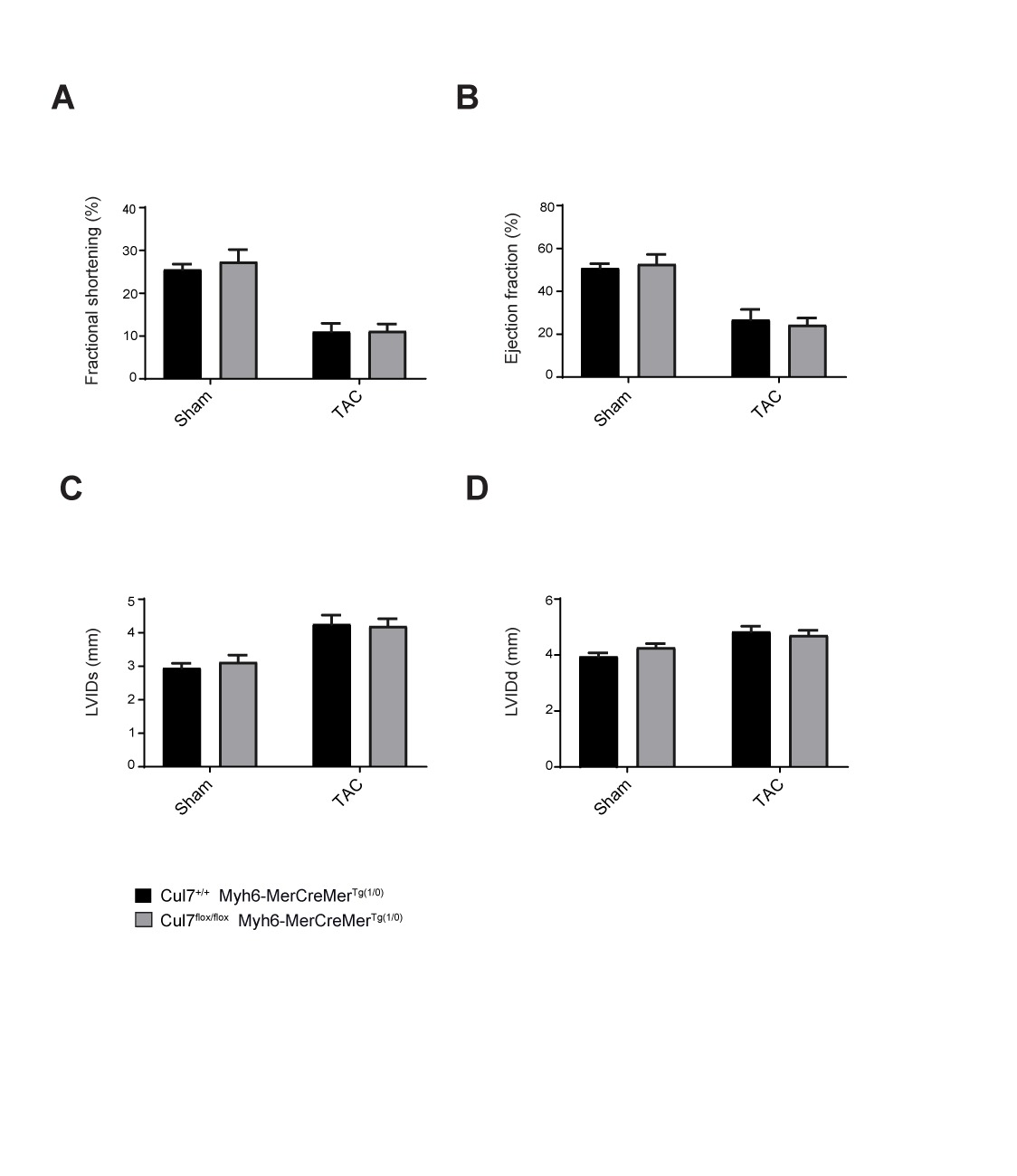

Supplement: S8 Fig — Lane 1–2 sham cohort, lane 3–4 TAC cohort. Functional assessment of (A) fractional shortening, (B) ejection fraction, (C, D) left ventricular inner diameter in systole and diastole (LVIDs resp. LVIDd) under basal conditions. n = 7–9 mice/group. Data are shown as mean ± SEM. *p<0.05, **p<0.01 (two-way ANOVA). (TIF) [file pone.0244096.s008.tif]

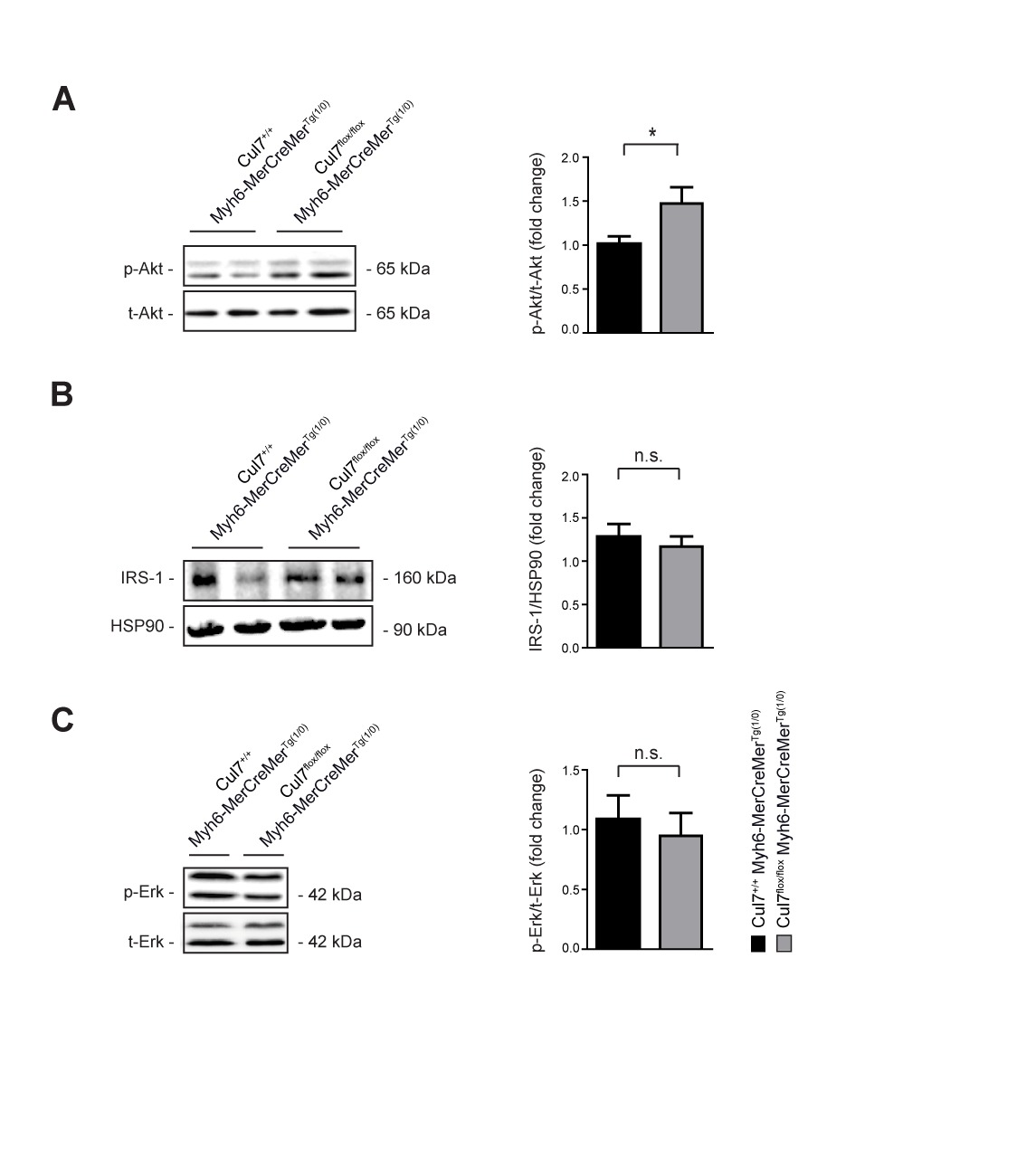

Supplement: S9 Fig — 8-week old mice were sacrificed 2 weeks after intraperitoneal injection of Tamoxifen. (A) Representative immunoblot showing IRS1 protein levels, HSP90 served as internal control (left); quantification (right). n = 7 mice/group. (B) Immunoblot analysis of p-AKT and total AKT. n = 7 mice/group. (C) Immunoblot analysis of p-ERK1/2 and total ERK1/2. n = 7 mice/group. All data are shown as fold change normalized to Cul7+/+ Myh6-MerCreMerTg(1/0) controls and expressed as mean ± SEM. Unpaired t-test. *p<0.05. (TIF) [file pone.0244096.s009.tif]

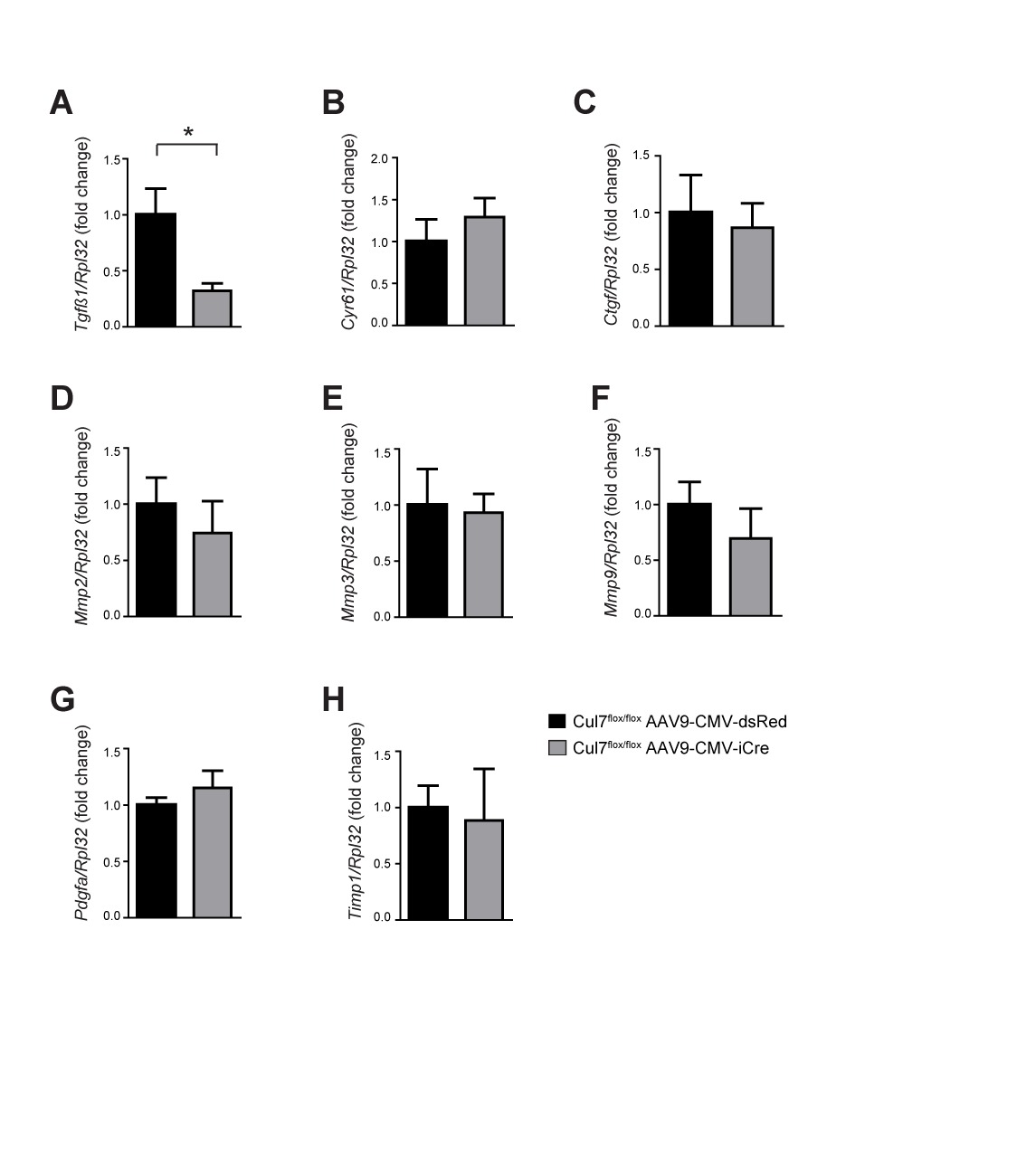

Supplement: S10 Fig — Hearts of 12-week-old Cul7flox/flox AAV9-CMV-iCre were harvested 3 weeks after TAC surgery, snap-frozen and analysed by quantitative RT-PCR. Relative gene expression was determined by normalization against Rpl32 as described in Materials and Methods and expressed as fold changes relative to control samples of Cul7flox/flox AAV9-CMV-dsRed mice (A-H). Values are means ± SEM, n = 3–6 mice/group. *p<0.05 (unpaired t-test). (TIF) [file pone.0244096.s010.tif]

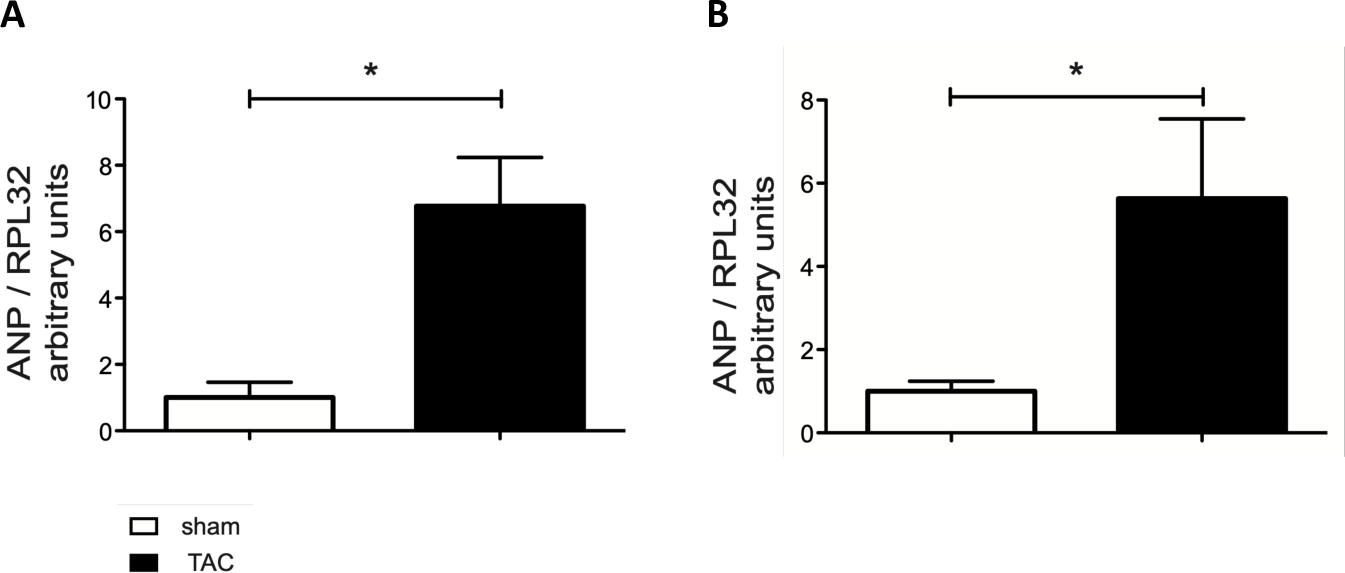

Supplement: S11 Fig — Quantification of ANP mRNA concentrations by realtime-PCR four (A) and six (B) weeks after transverse aortic constriction (TAC). Sham-treated littermates served as control. Rpl32 mRNA level served as internal controls. t- test; *: p < 0.05. (TIF) [file pone.0244096.s011.tif]

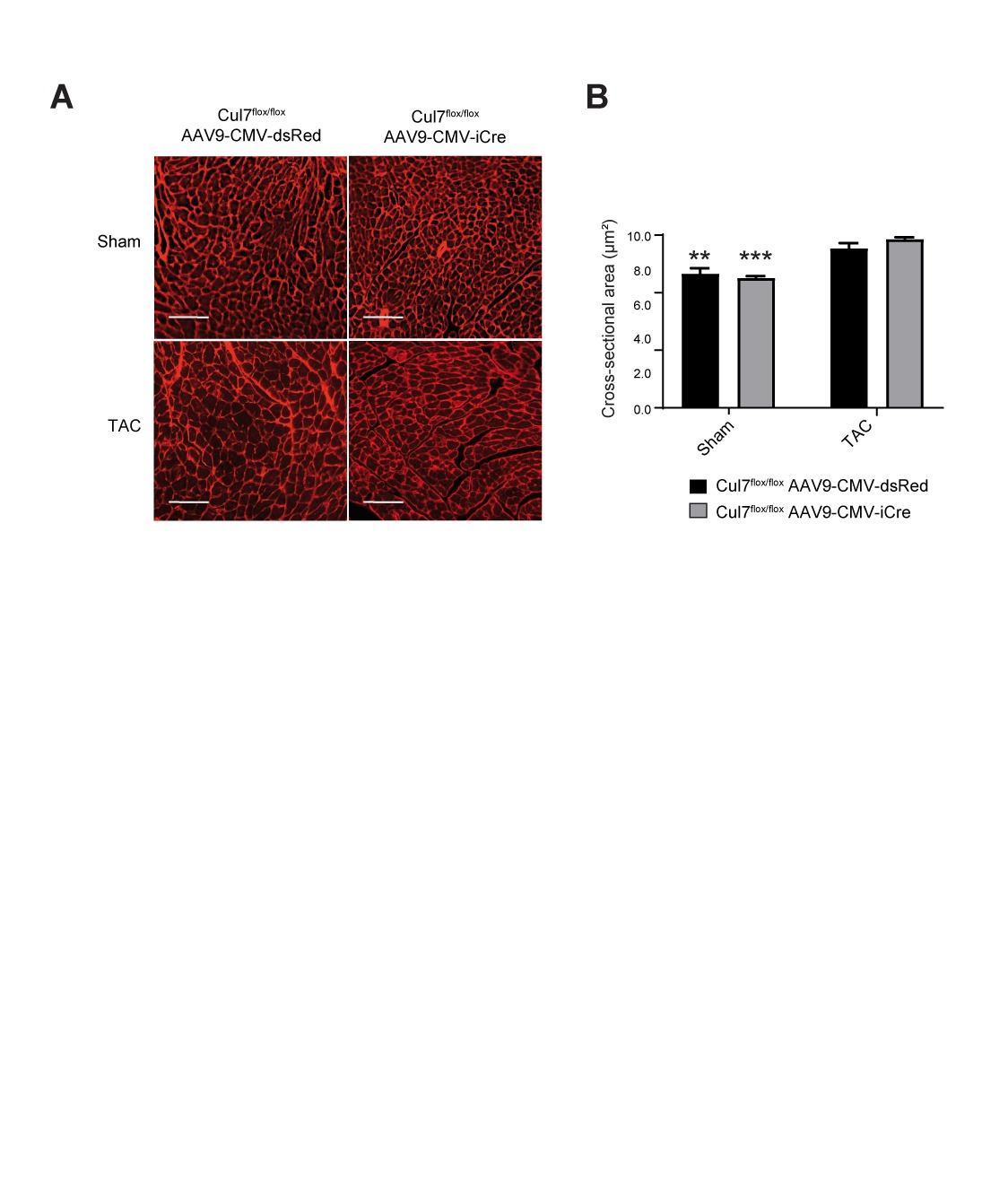

Supplement: S12 Fig — (A) Wheat germ agglutinin staining of representative myocardial sections of sham-operated animals (upper panel) and mice subjected to transverse aortic constriction (lower panel). (B) Quantification of cross sectional area. n = 5–6 mice/group; sham vs. TAC: ** P < 0.01, *** P < 0.001 (two-way ANOVA, Bonferroni post-test); scale bar = 100 μm. (TIF) [file pone.0244096.s012.tif]

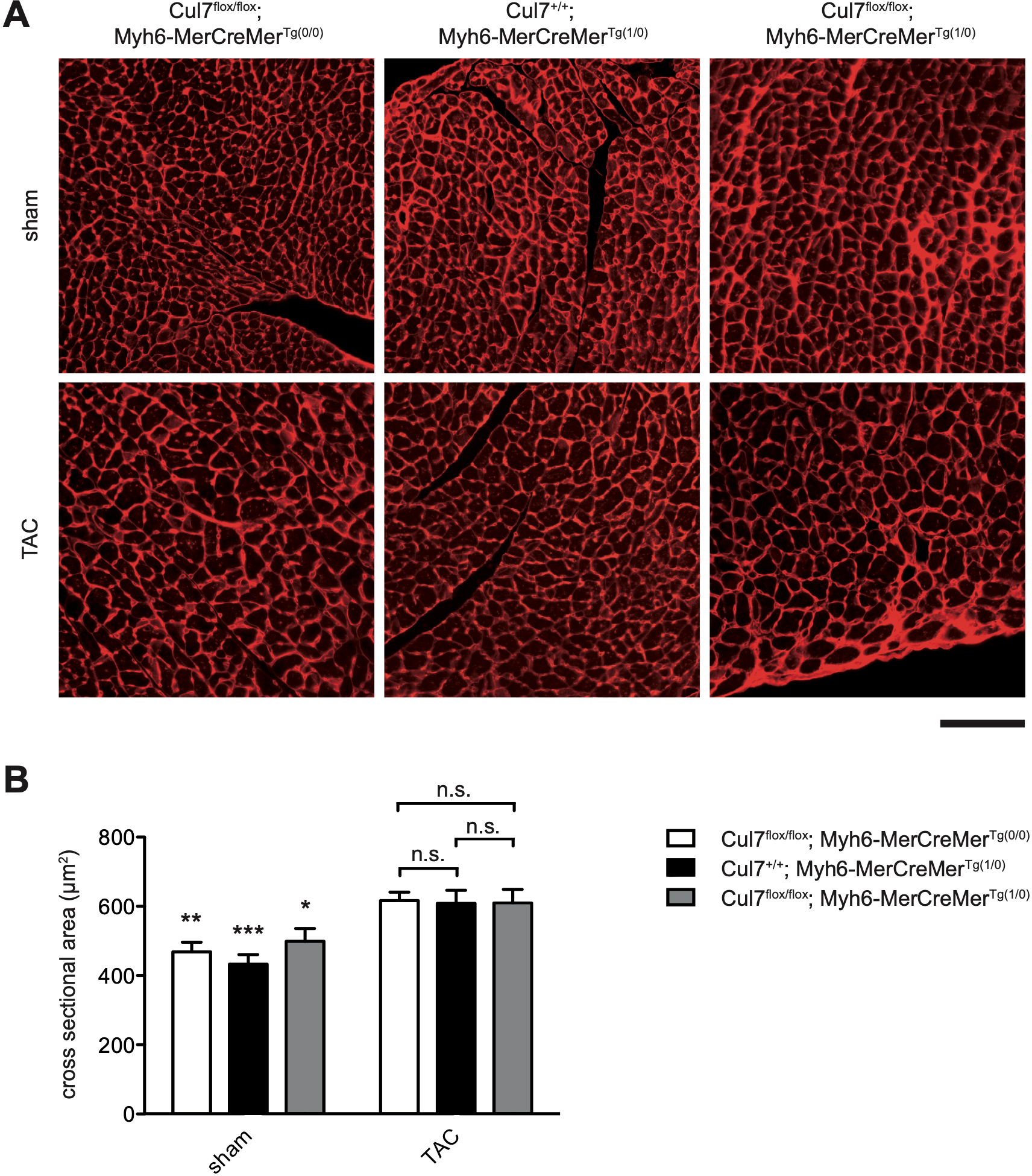

Supplement: S13 Fig — (A) Wheat germ agglutinin staining of representative myocardial sections of sham-operated animals (upper panel) and mice subjected to transverse aortic constriction (lower panel). (B) Quantification of cross sectional area. n = 5–6 mice/group; sham vs. TAC: * P < 0.05, ** P < 0.01, *** P < 0.001 (two-way ANOVA, Bonferroni post-test); scale bar = 100 μm. (TIF) [file pone.0244096.s013.tif]
