# Supplementary material for: Genetic ablation of Cullin-RING E3 ubiquitin ligase 7 restrains pressure overload-induced myocardial fibrosis
Source: PLoS One. 2020 Dec 22;15(12):e0244096. doi: 10.1371/journal.pone.0244096 (PMC7755222; doi:10.1371/journal.pone.0244096)
Supplement: S1 Table — (DOCX) [file pone.0244096.s014.docx]

**Table S1**

| Target | Direction | Primer Sequence (5’-3’) |
| --- | --- | --- |
| ANP | forward | GCTTCCAGGCCATATTGGAG |
| ANP | reverse | GGGGGCATGACCTCATCTT |
| Cyr61 | forward | ACCTCCTTGGATTCGATGCCT |
| Cyr61 | reverse | TGCCAAAGACAGGAAGCCTCT |
| Ctgf forward | forward | CGCCAAGCAGCTGGGAGAAC |
| Ctgf reverse | reverse | GGCGATTTTAGGTGTCCGGA |
| Cul7 forward | forward | CGGAATGGCTGTGCTGATG |
| Cul7 reverse | reverse | GCCTTGGATCCTCTGGTCAA |
| Mmp2 forward | forward | CGGAGATCTGCAAACAGGACA |
| Mmp2 reverse | reverse | CGCCAAATAAACCGGTCCTT |
| Mmp3 forward | forward | TGTCCCGTTTCCATCTCTCTC |
| Mmp3 reverse | reverse | TGGTGATGTCTCAGGTTCCAG |
| Mmp9 forward | forward | GCGTGTCTGGAGATTCGACTT |
| Mmp9 reverse | reverse | TATCCACGCGAATGACGCT |
| Pdgfa forward | forward | TGGCTCGAAGTCAGATCCACA |
| Pdgfa reverse | reverse | AGCCCCTACGGAGTCTATCTC |
| Rpl32 forward | forward | ACATCGGTTATGGGAGCAAC |
| Rpl32 reverse | reverse | GGGATTGGTGACTCTGATGG |
| dsAAVqSV40 fw2 * | forward | GCGACTCTAGATCATAATCAGCC |
| dsAAVqSV40 rw2 * | reverse | GCTGCAATAAACAAGTTAACAACAACA |
| Tgfb1 forward | forward | CTCCCGTGGCTTCTAGTGC |
| Tgfb1 reverse | reverse | GCCTTAGTTTGGACAGGATCTG |
| Timp1 forward | forward | CTATCCCTTGCAAACTGGAGA |
| Timp1 reverse | reverse | ACCTGATCCGTCCACAAACA |
