# Supplementary material for: Genetic ablation of Cullin-RING E3 ubiquitin ligase 7 restrains pressure overload-induced myocardial fibrosis
Source: PLoS One. 2020 Dec 22;15(12):e0244096. doi: 10.1371/journal.pone.0244096 (PMC7755222; doi:10.1371/journal.pone.0244096)
Supplement: S2 Table — (DOCX) [file pone.0244096.s015.docx]

**Table S2**

| **Assay** | **Genotype** | **# mice** | **Total Nuclei** | **ISEL +** | **Percent** | **P (Fisher’s)** |
| --- | --- | --- | --- | --- | --- | --- |
| Cardiomyocyte Apoptosis | MHC-Nlac / (-) | 13 | 78,525 | 51 | 0.065 |  |
|  | MHC-Nlac / MHC-p193dn | 7 | 48,505 | 2 | 0.004 | <0.001 |
| Cardiomyocyte S-phase | MHC-Nlac / (-) | 4 | 24,487 | 0 | 0.000 |  |
|  | MHC-Nlac / MHC-p193dn | 4 | 24,526 | 1 | 0.004 | n.s. |
