# Supplementary material for: Genetic ablation of Cullin-RING E3 ubiquitin ligase 7 restrains pressure overload-induced myocardial fibrosis
Source: PLoS One. 2020 Dec 22;15(12):e0244096. doi: 10.1371/journal.pone.0244096 (PMC7755222; doi:10.1371/journal.pone.0244096)
Supplement: Attachment — Submitted filename: Response to reviewers Anger_Scheufele 2020.docx [file pone.0244096.s016.docx]

**Editor`s comments**

**Please ensure that your manuscript meets PLOS ONE's style requirements, including those for file naming. The PLOS ONE style templates can be found at**

Done. The manuscript was revised to comply with PLOS ONE's style requirements.

**Please amend your current ethics statement to include the full name of the ethics committee that approved your specific study.**
 
Done. We have now included the following statement in the Methods section the manuscript as well as the “Ethics Statement” field of the submission form:

*“All animal studies were performed in accordance with the relevant guidelines and regulations and with approval of the responsible authorities (Regierung von Oberbayern, Munich, Germany;* permit number *55.2-1-54-2532-160-13).".*

**3. PLOS ONE now requires that authors provide the original uncropped and unadjusted images underlying all blot or gel results reported in a submission’s figures or Supporting Information files.**

Done. All original uncropped and unadjusted images underlying all blot or gel results reported in our manuscript`s figures or supporting Information files were posted to a public data repository and are publicly available at: URL

**Reviewers` comments**

**Reviewer #1:**

**The paper of Anger et al presents a series of experiments aimed at characterizing the role of CRL7 in cardiac remodeling and fibrosis. In particular, CM-specific knockout of Cul7 in two different mouse models results in significant reduction of interstitial fibrosis upon TAC, increased activation of PI3K/AKT axis, marked reduction of cellular apoptosis and significant downregulation of mRNA of Tgfb1. The results obtained from this study visibly suggest that CRL7 activation promotes fibrotic remodeling of the myocardium following pressure overload stress, and indicate Cul7 as a possible therapeutic target to counteract this process. The manuscript is well written and all the experiments are consistent with appropriate controls. Overall the results are assessed in a critical manner with some of the findings surprising but nonetheless interesting. Particularly intriguing is the evidence of the lack of functional cardiac improvement observed in Cul7-deficient mice, despite the remarkable reduction of fibrosis.**

We thank the reviewer for the detailed and insightful comments. We have made all efforts to improve the manuscript accordingly as outlined below.

**Although a deeper understanding of the mechanisms leading to this discrepancy is beyond the scope of this paper, I think that at least an analysis of biochemical and morphological markers of hypertrophy (fetal gene re-expression, myocytes cross sectional area) would be appropriate.**

In the revised manuscript, we have added additional data on biochemical and morphological markers of hypertrophy upon TAC. Atrial natriuretic peptide (ANP) mRNA expression was quantified by realtime-PCR of sham and TAC animal 4 and 6 weeks after intervention. The data was added to supplementary information as new **Fig. S11**.

In addition, the cardiac myocytes cross sectional area (CSA) of sham- or TAC-treated Cul7^flox/flox^AAV9-CMV-iCre or Cul7^flox/flox^AAV9-CMV-dsRed was measured. Furthermore, CSA of cardiac myocytes of sham and TAC-treated Cul7^+/+^ Myh6-Mer-Cre-Mer^Tg(1/0)^ or Cul7^flox/flox^ Myh6-Mer-Cre-Mer^Tg(1/0)^ was analyzed. These data show a robust induction of hypertrophy after TAC. We added these data as new **Figs. S12 and S13** to supplementary information.

The following paragraph was added to the Results section (page 11):

“*A robust induction of myocardial hypertrophy upon TAC was confirmed by realtime-PCR of atrial natriuretic peptide (ANP) and quantification of CM cross sectional area (****Figs. S11-13 appendix****).”*

**Additionally, the authors previously demonstrated that Cul7 downregulation improve the myocardium remodeling reactivates cardiomyocyte cell cycle and reduces hypertrophic cardiomyocyte growth in a mice model of permanent LAD ligation. In the present manuscript the authors highlight the anti-apoptotic action of Cul-7 ablation but did not mention possible cell-cycle activation mechanisms. Although the pathological models are quite different, these discrepancies should be discussed.**

Thank you for raising this important point. We have previously shown that expression of Cul7 MHC-1152stop and/or MHC-CB7 in mice resulted in reactivation of cardiomyocyte DNA synthesis in a myocardial infarction model, but not in uninjured hearts (Nakajima et al. Circ Res, 2004). We have now analyzed additional raw data of the 2004 study and compared cell cycle activity in MHC-Nlac (Cul7 +/+) vs. MHC-p193dn (Cul7 -/-) upon isoproterenol (ISO) treatment in a non-infarct setting. We observed no significant change in S-phase activity in ISO-treated WT vs. ISO-treated transgenic mice (0/24,487 for WT vs. 1/24,526 for transgenic, n = 4 mice each, p=n.s.; see also table below).

| **Assay** | **Genotype** | **# mice** | **Total Nuclei** | **ISEL +** | **Percent** | **P (Fisher’s)** |
| --- | --- | --- | --- | --- | --- | --- |
| Cardiomyocyte Apoptosis | MHC-Nlac / (-) | 13 | 78,525 | 51 | 0.065 |  |
|  | MHC-Nlac / MHC-p193dn | 7 | 48,505 | 2 | 0.004 | <0.001 |
| Cardiomyocyte  S-phase | MHC-Nlac / (-) | 4 | 24,487 | 0 | 0.000 |  |
|  | MHC-Nlac / MHC-p193dn | 4 | 24,526 | 1 | 0.004 | ns |

Thus, although cell cycle activity was not measured in the cardiac-restricted Cul7 null models employed in the present PLOS ONE manuscript, it is important to note that ISO treatment did not result in an increase in cardiomyocyte S-phase activity in transgenic mice expressing dominant-interferring Cul7 vs. WT mice. We therefore conclude that the marked reduction in cardiomyocyte apoptosis in these ISO-treated transgenic mice that we report in the present manuscript must occur independently of cardiomyocyte S-phase activity.

We added the following sentence to the Results section (page 14):

*“Of note, ISO treatment did not result in increased S-phase activity in transgenic mice expressing a dominant-interferring Cul7mutant in CM (****S2 Table****).”*

We added the following paragraph to the Discussion section (page 16):

*" Although cell cycle activity was not measured in the cardiac-restricted Cul7 null models employed in the present study, it is important to note that ISO treatment did not result in an increase in cardiomyocyte S-phase activity in transgenic mice expressing dominant-interferring Cul7 vs. WT mice. Thus, the marked reduction in cardiomyocyte apoptosis in these ISO-treated transgenic mice must occur independently of cardiomyocyte S-phase activity."*

In addition, we included the new cell cycle data as revised ***S2 Table****.*

**Reviewer #2:**

**This is a well done and interesting manuscript in which the authors conducted a series of experiments aimed at determining the role of Cullin-RING E3 ubiquitin 1 ligase 7 in cardiac hypertrophy. They have generated models of inducible genetic deletion in mice and subjected the animals to pressure overload induced hypertrophy to determine the effects on cardiac function under stress. Results show that this gene controls fibrosis as its absence ameliorate cardiac function, decreasing the expression of collagen by many folds as well as cardiac fibrosis and apoptosis.
I have no major questions, only some clarifications which could be added to the discussion.**

We thank the reviewer for the detailed and insightful comments. We have made all efforts to improve the manuscript accordingly as outlined below.

**How these results could be exploited for therapeutics is not clear. The authors may speculate on this.**

We thank the reviewer for raising the translational implications of our findings. Proof-of-concept studies have shown that CRLs are druggable targets. For instance, suramin, an antitrypansomal drug that also possesses antitumor activity, was identified through a high-throughput screen as inhibitor of ubiquitination and shown to interfere with the binding of Cdc34 to cullin proteins (Wu et al. PNAS 2016). It was shown that suramin potently inhibits the activity of a variety of CRL complexes containing cullin 2, 3, and 4A. As our results demonstrate that inactivation of CRL7 results in amelioration of cardiac fibrosis, it is tempting to speculate that small molecules that inhibit CRL7 may provide a novel therapeutic strategy for the treatment of cardiac fibrosis.

We now included the following paragraph in the revised discussion section (page 19):

*“Proof-of-concept studies have shown that CRLs are druggable targets. Wu et al. recently reported the identification of the small molecule drug suramin as inhibitor of CRLs that contain cullin 2, 3, and 4A (PMID: 27001857). Whether targeting CRL7 by small molecules will provide a novel strategy to impede excess fibrosis in the heart awaits future investigation.”*

**Is there a link between production of collagen and apoptosis? Can the authors speculate also on the effects of AKT activation, which most probably plays an anti-apoptotic effect and its potentially negative effect on autophagy?**

In the present manuscript we show that inactivation of CRL7 in CM results in increase AKT signaling, reduced CM apoptosis and cardiac fibrosis. It is tempting to speculate that the anti-apoptotic effect of CRL7 inactivation in CM leads to a decreased need for collagen synthesis as compensatory or replacement mechanism in the context of myocardial remodeling after TAC.

We discussed this issue now on page 17: “It is thus tempting to speculate that CRL7-mediated regulation of PI3K/AKT signalling contributes to CM survival, thereby preventing replacement fibrosis”.

**Since the KO has been performed in the cardiomyocyte compartment, is there a cross talk between the cardiomyocyte and the fibroblasts, which should be the primary source of collagen in the heart?**

We thank the reviewer for this interesting question. Fibrosis is a hallmark of most myocardial pathologies and it is increasingly appreciated that the cellular microenvironment and cross-talk between CMs and CFs play a critical role in fibrogenesis (reviewed in Martin ML, Blaxall BC. J Cardiovasc Transl Res. 2012.). CM death is often the triggering event responsible for the initiation of fibrosis in the context of reparative cardiac fibrosis. In addition, reactive fibrosis , characterized by the absence of cell death, is was shown to stimulate different cell types within the myocardium, thereby producing fibrous tissue directly or indirectly by secreting profibrotic mediators (reviewed in González et al. J Am Coll Cardiol, 2018).

To further discuss this aspect we have now included the following sentence in the Discussion section of the revised manuscript (page 18):

*“However, we cannot rule out that CRL7 also affects other pro-fibrotic pathways independent of cell death.”*

**There are in the literature manuscripts demonstrating that AKT activation favors the capacity of the heart to cope with pressure overload stress. This effect is mediated also by an increased inotropic and, at the level of the myocyte, lusotropic effect. Have the authors tried to analyze cardiac function on isolated cardiomyocytes? Could they speculate on that?**

We thank the reviewer for this interesting point. We have not investigated the cardiac function on isolated cardiomyocytes. To discuss the effect of AKT signaling on CM contractility, we have now included the following sentence in the Discussion section of the revised manuscript (page 19):

*“In addition, loss of regulated AKT activity upon genetic ablation of CRL7 may have affected cardiac function, as balanced AKT signaling is essential for contractility and the response to pathological stress ^46^.”*

**5. AKT activation seems to decrease the level of inflammation which accompanies pressure-overload HF; due to the link between inflammation and fibrosis, this should be mentioned in the discussion.**

We thank the reviewer for this interesting point. We added the following paragraph to the Discussion section (Page 18):

“*In addition, PI3K/AKT signaling pathways regulate key events in the inflammatory response to damage ^44^. It is therefore tempting to speculate that the observed effect of CRL7 ablation on cardiac fibrosis is partly mediated by PI3K/AKT-regulated inflammation and its downstream effects on fibrogenesis*.”
